# Key Hormonal Components Regulate Agronomically Important Traits in Barley

**DOI:** 10.3390/ijms19030795

**Published:** 2018-03-10

**Authors:** Marek Marzec, Ahmad M. Alqudah

**Affiliations:** 1 Department of Genetics, Faculty of Biology and Environmental Protection, University of Silesia, 40-032 Katowice, Poland; 2 Department of Physiology and Cell Biology, Leibniz Institute of Plant Genetics and Crop Plant Research (IPK), 06466 Stadt, Seeland, Germany; 3 Research Group Resources Genetics and Reproduction, Genebank Department, Leibniz Institute of Plant Genetics and Crop Plant Research (IPK), 06466 Stadt, Seeland, Germany

**Keywords:** agronomical traits, barley, genome-wide association studies, phytohormones, plant architecture, spikelet development

## Abstract

The development and growth of plant organs is regulated by phytohormones, which constitute an important area of plant science. The last decade has seen a rapid increase in the unravelling of the pathways by which phytohormones exert their influence. Phytohormones function as signalling molecules that interact through a complex network to control development traits. They integrate metabolic and developmental events and regulate plant responses to biotic and abiotic stress factors. As such, they influence the yield and quality of crops. Recent studies on barley have emphasised the importance of phytohormones in promoting agronomically important traits such as tillering, plant height, leaf blade area and spike/spikelet development. Understanding the mechanisms of how phytohormones interact may help to modify barley architecture and thereby improve its adaptation and yield. To achieve this goal, extensive functional validation analyses are necessary to better understand the complex dynamics of phytohormone interactions and phytohormone networks that underlie the biological processes. The present review summarises the current knowledge on the crosstalk between phytohormones and their roles in barley development. Furthermore, an overview of how phytohormone modulation may help to improve barley plant architecture is also provided.

## 1. Introduction

**Figure 1:**
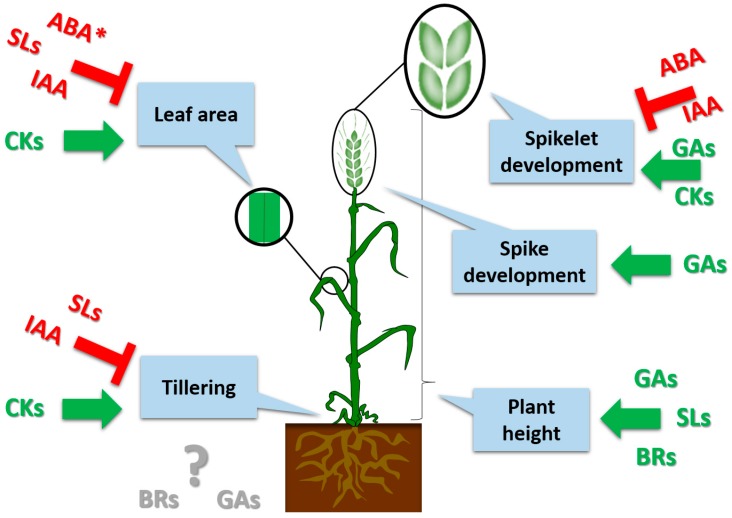
Role of phytohormones in plant development traits. Green indicates a positive effect and red indicates an inhibitory effect of a hormone on a trait; grey indicates inconsistent experimental data; a star * indicates that the inhibitory effect of ABA on leaf area was observed only under drought stress. ABA—abscisic acid, BRs—brassinosteroids, CKs—cytokinins, GAs—gibberellic acids, IAA—auxin, SLs—strigolactones.

Manipulating the architecture of agronomic traits in cereals has a clear impact on plant adaptation to changing environmental conditions and improvement of the grain yield. Currently, much research is being directed towards the genetic dissection of the architecture and yield of plants so that targeted traits can be achieved more efficiently. Climate change and a growing human population demand that new crop varieties have to be better adapted to the local environmental conditions, while still producing sufficient high-quality yields [1]. The aim of the crop ideotype concepts that were proposed during the 1960s and 1970s was to enhance grain yield by modifying crop architectural traits through cereal breeding programmes [2]. The concept of ideotypes in breeding programmes is based on understanding the morphological, anatomical, and genetic traits and using this knowledge to develop plants that will produce enhanced grain yields under specified conditions [2]. The most important factors that define an ideotype are spike architecture, plant height, the number of fertile tillers, leaf blade area and phase duration [3], all of which are controlled by phytohormones (Figure 1). One of the first proposed barley ideotypes indicated that under constant drought stress, the ratio between the vegetative and generative periods and a smaller leaf area were crucial traits, whereas under water-sufficient conditions, the canopy profile was one of the most important traits influencing grain yield [4]. Because of climate change, increasing consumption and changes in human diets, crop ideotypes need constant improvement [1,5]. The greatest challenge in barley ideotypes is to select a targeted trait that is both heritable and that can be adjusted for a specific environment.

Most of what we know about the role of phytohormones in the growth and development of *Hordeum vulgare* L. (barley) is based on research from other grass species such as *Oryza sativa* L. [6] and *Zea mays* L. (maize) [7,8]. However, recent studies have identified a large set of genes that play a crucial role in the biosynthesis and/or signalling cascades of hormones, such as brassinosteroids (BRs), cytokinins (CKs), gibberellins (GAs) and strigolactones (SLs) [9,10,11]. These results permit the key hormonal components that regulate the shoot and spike architecture in barley to be predicted.

A powerful tool that is used to analyse and identify the genetic factors that control the complex architectural traits at a population level is the so-called genome-wide association studies (GWAS). GWAS has become a widely accepted approach in the study of plants. Basically, it detects associations between genotypic variations and differences in phenotypes for a given population using the appropriate statistical model. During the past few years, GWAS has enabled scientists to produce high-resolution genetic maps of several large-genome crops that have facilitated the identification of the genes that underlie the natural phenotypic variation of agronomic–architectural traits. In barley, GWAS has been used to identify single nucleotide polymorphism (SNP) markers that are associated with the root architecture [12], tillering [10], plant height [10], leaf area [11,13,14] and spike architecture [9,15]. Moreover, GWAS has also shown great potential to unravel the genetic background of architectural traits of other crop plants [6,16]. Alqudah et al. found that many of the QTL (quantitative trait loci) were precisely associated with known plant stature-related phytohormone genes, such as leaf area, tillering and plant height [10,11]. Therefore, using such a robust approach can help answer genetic and biological questions about complex traits, for instance, to discover the phytohormone genes that underlie agronomic traits.

Recent studies have indicated that the most important traits related to increased grain yield in barley include: (1) an extended phase critical for spike development, including increasing the number of spikelets and increasing spikelet survival, (2) a higher water-use efficiency and (3) abiotic stress tolerance, which is related to (4) a greater maximum root depth [3]. Here, we review the recent advances in understanding the molecular mechanisms of phytohormones in the architecture and yield of barley.

## 2. Hormonal Regulation of Agronomically Important Traits in Barley

### 2.1. Roles of Phytohormones in Plant Height 

Combined breeding, genetic and physiological approaches have demonstrated the importance of hormones in improving plant stature, adaptation and yield. A well-known example is the reduction in plant height during the Green Revolution in order to increase lodging resistance and limit yield [17]. Three main hormonal players regulate plant height: BRs, GAs and SLs. In barley, disorders in the biosynthesis or signalling pathways of these hormones, which result in lower production or insensitivity to a hormone that is produced, leads to dwarf or semi-dwarf forms [18,19]. Because GAs promote both cell proliferation and cell division, mutants with a decreased production of GAs or that are insensitive to GAs have shorter culms [20]. BRs also increase cell elongation and it has been postulated that they regulate cell growth, i.e., via the promotion of GA accumulation in rice [21]. However, in the case of mutants with deficient SL biosynthesis or reduced signalling during growth, this might not only be related to the larger number of outgrowing tillers. Additionally, a positive effect of SL on the number of cells has been observed and it has been proven that SLs stimulate internode elongation independently of GAs [22]. Mutants with short culms have already been used successfully in breeding programmes of barley, wheat and rice [23,24,25]. However, in the rush to adapt modern crop plants to changing environmental conditions, the demand for new dwarfing alleles remains unabated. One reason is the fact that the phenotypic effect of some alleles may depend on the environmental conditions. Although it was shown for one of the *HvBRI1* alleles (*uzu*) that a higher temperature (26 °C) resulted in a more drastic dwarf phenotype compared with a lower temperature (14 °C), this effect has not been observed for the other known alleles of *HvBRI1* [19]. Among the QTL for plant height in barley that have recently been identified, one that is involved in BR biosynthesis, *DWARF4* (*HvD4*) (Table 1) [10], has not yet been described for this species [19]. The other gene *HvCPD*, which encodes the protein that is involved in BR biosynthesis, was previously characterised in barley. A mutation in *HvCPD* results in a semi-dwarf phenotype of the mutant [19] but no information about any additional effects of this mutation, i.e., on tillering or leaf area, has been described. 

### 2.2. The Role of Phytohormones in Tillering

One of the most important traits that have an influence on yield in cereals is the number of fertile tillers, which is controlled by environmental, genetic and hormonal factors. Recently, significant progress has been made in uncovering the hormonal regulation of shoot branching in grasses [26,27]. For a long time, auxin (IAA) [28] and CKs [29] were considered to be key components in regulating tillering. In cereals such as rice, polar export of IAA from axillary buds is necessary to promote bud outgrowth and local treatment with IAA inhibits tillering and decreases the level of CKs [30]. CKs are positive regulators of branching that act antagonistically to IAA and promote tiller bud outgrowth in barley [29] and rice [31]. With the discovery of the negative role of SLs in regulating the tiller number in highly branching mutants in rice, it became clear that besides IAA and CKs, SLs represent a third class of hormones that regulate the tiller number [32]. A recently identified barley mutant in the SL receptor confirmed that SLs inhibit the export of IAA from tiller buds, thus preventing tiller outgrowth [18]. GWAS studies have revealed that a hitherto functionally unknown gene in the QTL for tillering in barley—*HvD10*—encodes carotenoid-cleavage dioxygenase, which is involved in SL biosynthesis [33]. Based on observations in rice [34] and barley [35], GAs have also been hypothesised to regulate branching. Studies in rice have shown that GAs regulate the biosynthesis of SLs [36], thus indicating that both hormone classes may act together in tillering regulation [37]. This hypothesis was confirmed by a GWAS analysis showing that the group of genes that is associated with tillering is dominated by those that are involved in GA biosynthesis and signalling (Table 1) [10]. The role of BRs in tillering in cereals remains unclear. While Tong et al. [38] reported that elevated BR levels are related to a lower number of tillers in rice, Wu et al. [39] found a positive correlation between the expression of the genes that are involved in BR biosynthesis and increased tillering. A decrease in the tiller number was also found in the barley BR-insensitive mutant *uzu* [40], but this effect was not observed in the mutant allelic to *uzu* [41] and other BR mutants in barley [19]. Interestingly, the genes encoding the proteins for BR biosynthesis and signalling pathways comprise the second largest group that is associated with tillering in GWAS studies (Table 1) [10]. A more detailed analysis of individual mutants in relation to tillering is necessary in order to answer the questions about the role of BRs in this developmental process.

### 2.3. Phytohormone Regulation of Spike and Spikelet Development and Fertility

**Figure 2:**
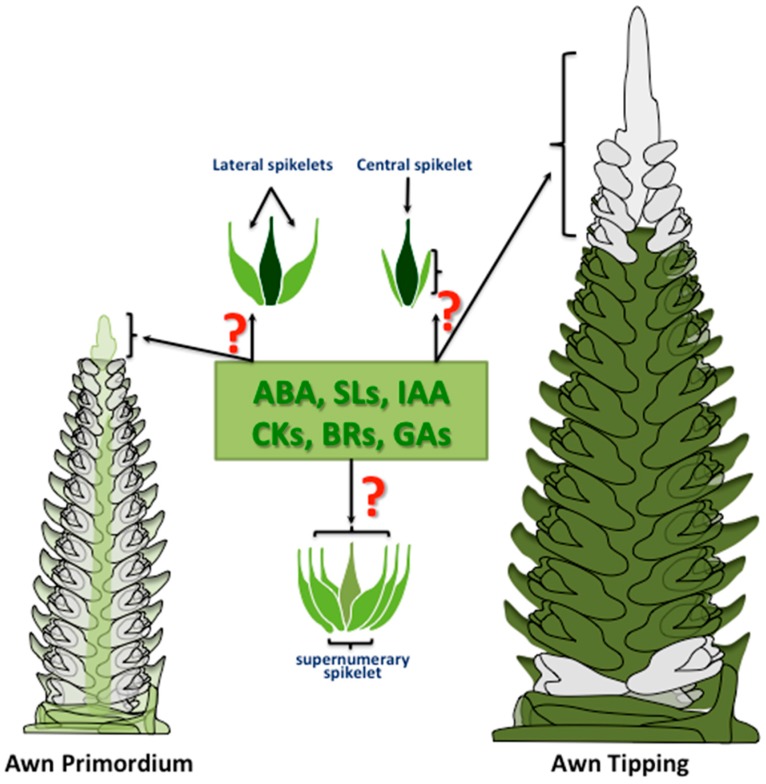
Role of phytohormones in spikelet and spike development-related traits. Question marks denote that the impact of phytohormones remains undiscovered. ABA—abscisic acid, BRs—brassinosteroids, CKs—cytokinins, GAs—gibberellic acids, IAA—auxin, SLs—strigolactones.

Phytohormones are closely linked with the transition phase and organ development, especially during the reproductive phase, for instance, the development of spikelets and the floral organs. Spike development is mainly influenced by GAs since treatment with GAs accelerates spike development in wheat and induces the expression of the floral meristem identity genes [42]. When wheat plants are transferred from short- to long-day conditions, the genes that are involved in GA biosynthesis become upregulated in the apices [43]. *REDUCED HEIGHT* (*RHT*), one of the genes used during the Green Revolution, encodes a protein from the DELLA family, which are negative regulators of GA signalling. A gain-of-function mutation in *RHT* not only results in a semi-dwarf phenotype but also an increase in spikelet fertility [44]. GAs promote heading in spring barley, which demonstrates the importance of GAs in adaptation and yield improvement through reducing plant height and improving lodging resistance, which are correlated with higher yields and quality traits [45]. It has long been hypothesised that other hormones also play important roles in spike and spikelet/floret development. Wang et al. [46] reported on the effects of hormones injected into the leaf sheath around young spikes on wheat floret development and grain set. Whereas the injection of CKs promoted the development of wheat florets, thus increasing the number of fertile florets and the grain set, injections of IAA, GAs and abscisic acid (ABA) inhibited floret development [46]. Zheng et al. [47] found that wheat floret development and grain setting were improved by applying synthetic CKs (6-benzylaminopurine, 6-BA). In rice, GAs and kinetin (6-furfuryl amino purine) enhanced spikelet growth and development and increased the grain yield on all rice branches, while IAA influenced only the distal branches [48]. The endogenous hormone levels in wheat are stage dependent. In the spike and anther developmental phases, the level of ABA and GAs is decreased, which might improve fertile florets and grain set [49,50]. Recently, it was shown that the gradients of IAA and CKs are not distributed homogeneously during spikelet development in barley. While the concentration of IAA is highest in the basal region, declining towards the apical region, the concentration of CKs display a reverse gradient, declining towards the basal region [9]. In plants carrying a mutation in the *Six-rowed spike2* (*vrs2*.e) encoding SHORT INTERNODES (SHI) transcription factor, IAA and CK gradients are absent and spike development is disordered. Spikes of wild-type plants also have a higher concentration of GAs than the vrs2.e mutants, where it has a role in shaping the spike architecture by controlling spikelet development and fertility [9]. In rice, Cai et al. [51] discovered the role of jasmonic acid (JA) in regulating the determinacy of rice floral meristem and spikelet morphogenesis. *Extra Glume 1* (*EG1*) and *EG2* mutants show changes in spikelet morphology as well as the floral organ identity and number (Table 2). EG1 acts in JA biosynthesis and EG2 is a JA signalling repressor that produces a defective floral meristem determinacy (Table 2). The role of phytohormones in spikelet development and fertility might be applicable to other grasses. Such work provides the basis for improving crop yield. Therefore, whether hormonal patterns are the cause or the consequence of spikelet fertility in cereal spikes remain to be explored (Figure 2).

Alqudah and Schnurbusch [52] found that the phase between the emergence of awn primordium and awn tipping is the most critical phase for spikelet abortion where anthesis or fertilisation occurs [53]. It is a critical yield-determining trait that is genetically controlled and is influenced by environmental factors [52]. Therefore, proper development of the floral organs such as the anther during this phase is essential for improving spikelet survival. Phytohormones play an important role in regulating the development of the floral meristem. Thus, understanding how a phytohormone regulates spikelet development and fertility is crucial for improving grain yield. Studies in rice have shown how unregulated GA signalling leads to defects in the formation and development of the floral organs that in turn increase the sterility of spikelets. The DELLA protein *Slender rice 1* (*SLR1*) acts as a negative regulator of downstream genes in GA signalling and plays a key role in the development of the floral organs (Table 2), where a deficiency of *SLR1* causes spikelet fertility phenotypes [54]. For instance, although a gain-of-function in an *Slr1-d3* mutant produces normal pistils and stamens, semi-fertile phenotypes are caused by a low pollen viability [54]. Loss-of-function in the *SLR1* mutant *slr1-1* clearly shows sterile phenotypes [55]. 

In addition, the GA-insensitive rice mutant *gid1-4* displays an abnormality in the development of organs during anther development [59], thus indicating that GID1 is necessary for the structure of the anthers. The mutants of *EG1* and *EG2* (i.e., *eg1-3* and *eg2-1D*), which are involved in JA biosynthesis and signalling, show defects in the spikelet organ. For example, the mutants develop extra glume-like structures, the palea exhibit lemma-like, the lodicules in both mutants are transformed into glume-lodicule mosaic structures and there are decreased numbers of stamens and pistils, which suggest a role of JA in floral meristem determinacy and floral organ identity [51]. 

Youssef et al. [9] revealed that barley spikes (i.e., the two-rowed cv. Bowman) have higher concentrations of GA20ox (GA 53, GA44 and GA19) in the central spike parts during the white (WA) and green anther (GA) developmental stages compared with BW-NIL (*vrs2.e*). IAA has a high concentration in the basal part of a spike at the WA and GA stages that is in an antagonistic trend to cytokinin (*t*-Zeatin) [9]. Although barley spikes have an indeterminate number of rachis nodes, each node produces three single spikelets—one central and two lateral (e.g., *VRS1*)—or produces many spikelets (supernumerary spikelets) per node (e.g., *VRS2* and *VRS4*). Because the lateral spikelets are sterile in the two-rowed barley (*VRS1*), one can suppose that the phytohormones might play a role in the fertility of the lateral spikelets, spikelet development, supernumerary spikelet development and shaping the spike architecture (Figure 2). Interestingly, because the spatiotemporal patterns of a phytohormone are in synchrony with spikelet fertility and abortion, understanding the pattern of a phytohormone along the spike (basal, central and top), a specific spike organ (e.g., anther) and spikelet sections (lateral vs. central) during this critical phase (awn primordium and awn tipping) is important for regulating the yield processes such as spikelet abortion in cereal crops.

### 2.4. Phytohormone Regulation of the Leaf Area

The last architectural trait, which is also important for adaptation and yield improvement, is the leaf area, which is strictly related to photosynthesis [60]. The size and shape of a leaf, the leaf position and exposure to sunlight are associated with photosynthetic efficiency and hence with the amount of assimilates that are produced by the plant [61,62]. Plants with a higher supply of photoassimilates can develop more fertile tillers, feed more spikes and increase spikelet survival, which subsequently improves yield. Because the flag leaf is the main source of carbohydrates for the developing spikes [63] in cereals, the leaf area plays an important role in the regulation of plant development in both the vegetative and reproductive phases. Heavily branched mutants usually have more leaves and hence a larger leaf area as was observed in the SL-insensitive barley mutant *hvd14.d* [18]. However, it should be mentioned that a larger leaf area is related to faster transpiration and water loss under drought stress. A GWAS analysis has revealed nine genes that are associated with the leaf area in barley [11]. Three of them are related to GAs, three to BRs and three to SLs (Table 1) [10,11]. Some of these genes are specifically associated with this trait, which indicates that GAs, BRs and SLs might regulate the leaf area independently from traits such as branching. In addition, this analysis provides promising candidate genes for further analysis of the promotion of leaf development in barley [11]. However, it has to be stressed that one of the SL genes—a homologue of *MAX2*—is also involved in the signalling pathways of other phytohormones [37]. Hence, the hormonal regulation of the development of the leaf area is still unclear and more advanced molecular genetic analyses are required in order to understand the mechanisms that underlie this process.

## 3. Conclusions and Remarks

Population growth and climate change are the driving forces behind the incessant efforts to improve crop plants. In order to maintain food security, crop adaptation, architecture and grain yield need to be optimised. In order to achieve this aim, it is crucial to understand the factors that regulate these important plant features. This is one reason why the functional analysis of individual genes is necessary to evaluate their precise role. To speed up the process of gene selection for breeding programmes, large-scale analyses such as GWAS are useful for identifying the genes that are related to specific traits. Recently, published results have indicated that mutants affected during BR or GA biosynthesis and signalling may be considered as a source of the alleles that promote tillering. Knowledge about gene function and gene position within a genome permits the direct and accurate screening of barley varieties and mutant collections, thus avoiding costly and lengthy phenotypic studies. This review contributes to establishing a basis for further molecular physiology and genetic work within the context of barley plant architecture that is based on hormonal effects. 

## Figures and Tables

**Table 1:** List of the barley genes that are associated with agronomically important traits as identified by genome-wide association studies (GWAS) (according to [10,11]).

No.	Chr.	cM (POP SEQ)	Gene	Barley High Conf. Gene	Contig Identifier	Trait	Hormone
1	1H	5.38	*BRASSINOSTEROID-6-OXIDASE (HvBRD1)*	AK372445	morex_contig_244330 CAJW010244330	Tillering	BRs
2	1H	55.52	*GIBBERELLIN INSENSITIVE DWARF1 (HvGID1)*	AK356665	morex_contig_137029 CAJW010137029	Tillering	GAs
3	1H	94.75	*GIBBERELLIN 20 OXIDASE 4 (HvGA2ox4)*	MLOC_13981.1	morex_contig_1566970 CAJW011566970	Tillering and leaf area	GAs
4	2H	58.78	*GIBBERELLIN-INSENSITIVE DWARF 2 (HvGID2)*	MLOC_61457.1	morex_contig_41142	Tillering	GAs
5	2H	59.91	*DWARF 11 (HvD11), CYTOCHROME P450 724B1*	AK371371	morex_contig_45000 CAJW010045000	Tillering	BRs
6	3H	44.26	*DWARF 2 (HvD2), CYTOCHROME P450 90D2*	MLOC_62829.1	morex_contig_47012 CAJW010047012	Tillering	BRs
7	3H	46.03	*GIBBERELLIN 3 OXIDASE 2 (HvGA3ox2)*	MLOC_12855.1	morex_contig_51542 CAJW010051542	Tillering	GAs
8	3H	46.03	*DWARF 18 (HvD18)*	MLOC_12855.1	morex_contig_51542 CAJW010051542	Tillering	GAs
9	3H	51.34	*BRASSINOSTEROID INSENSITIVE 1 /*SEMIBRACHYTIC/*Dwarf61* (*HvBRI1/ uzu1 HvD61/)*	MLOC_5176.2	morex_contig_58772 CAJW010058772	Tillering and leaf area	BRs
10	3H	62.93	*MORE AXILLARY BRANCHES 4/ CAROTENOID CLEAVAGE DIOXYGENASE 8 /DWARF 10 (HvMAX4/CHvCD8/ HvD10)*	MLOC_66551.1	morex_contig_51744 CAJW010051744	Tillering and leaf area	SLs
11	3H	64.16	*GIBBERELLIN 20 OXIDASE 1 (HvGA2ox1)*	AK364775	morex_contig_2550522 CAJW012550522	Tillering and leaf area	GAs
12	3H	106.02	*GIBBERELLIN 20 OXIDASE 3 (HvGA20ox3)*	MLOC_66389.1	morex_contig_51490 CAJW010051490	Tillering	GAs
13	4H	59.63	*DWARF 4 (HvD4)*	AK355174	morex_contig_61948 CAJW010061948	Plant height	BRs
14	5H	44.02	*BRASSINOSTEROID C-23 HYDROXYLASE (HvCPD)*	MLOC_10658.1	morex_contig_1559549 CAJW011559549	Tillering, plant height and leaf area	BRs
15	5H	46.59	*DWARF 53 (HvD53)*	AK372211	morex_contig_244827 CAJW010244827	Leaf area	SLs
16	5H	47.22	*BRITTLE CULM12/ GIBBERELLIN-DEFICIENT DWARF 1 (HvBC12/GGD1)*	AK373790	morex_contig_45441 CAJW010045441	Tillering	GAs
17	5H	80.8	*DWARF RICE WITH OVEREXPRESSION OF GIBBERELLIN-INDUCED GENE (HvDOG)s*	AK359310	morex_contig_1575121 CAJW011575121	Tillering	GAs
18	7H	29.95	*MORE AXILLARY BRANCHES 2 (HvMAX2)*	MLOC_4044.5	morex_contig_134615 CAJW01013461	Leaf area	SLs
19	7H	77.4	*DWARF 35 (HvD35), CYTOCHROME P450 701A6*	AK369327	morex_contig_1575857 CAJW011575857	Tillering and leaf area	GAs
20	7H	140.65	*BRASSINOSTEROID DEFICIENT DWARF 2/ DIMINUTO, DWARF1 (HvBRD2/HvDIM/HvDWF1)*	MLOC_52405.2	morex_contig_37512 CAJW010037512	Tillering and leaf area	BRs

ABA—abscisic acid, BRs—brassinosteroids, CKs—cytokinins, GAs—gibberellic acids, IAA—auxin, SLs—strigolactones.

**Table 2:** Phytohormone-related genes involved in spikelet development and fertility.

Chr	Physical pos.	Gene	Barley HC Gene	Annotation	Accession	GO Term	Function	Hormone
3H	627490751-627493520	*EXTRA GLUME 1 EG1*	*HORVU3Hr1G089140*	Phospholipase A1-II 1	24647160	GO:0006629	Regulation of spikelet development [51]	JA [51]
2H	725648938–725655525	*EXTRA GLUME 2 EG2*	*HORVU2Hr1G112360*	jasmonate-zim-domain protein 12	AK069326	none	Regulation of spikelet development [51]	JA [51]
4H	16668801–16671743	*SLENDER 1 SL1*	*HORVU4Hr1G006930*	DELLA protein	AB262980	none	Regulation of floral development and spikelet fertility	GAs
1H	441473716–441477633	*GIBBERELLIN INSENSITIVE DWARF1 GID1*	*HORVU1Hr1G060810*	Gibberellin receptor GID1	AK074026	GO:0008152, GO:0016787		GAs
5H	564406197–564410417	*Six-rowed spike 2 VRS2*	*HORVU5Hr1G081450.1*	SHI-related sequence 5	KX601696.1	none	Regulation of spikelet development and fertility [9], awn elongation and pistil shape [56]	IAA [57], GAs [58]

GAs—gibberellic acids, IAA—auxin, JA—jasmonic acid.

## References

[1] Ray D.K., Gerber J.S., MacDonald G.K., West P.C. (2015). Climate variation explains a third of global crop yield variability. Nat. Commun..

[2] Donald C.M.T. (1968). The breeding of crop ideotypes. Euphytica.

[3] Tao F., Rötter R.P., Palosuo T., Díaz-Ambron C.G.H., Mínguez M.I., Semenov M.A., Kersebaum K.C., Nendel C., Cammarano D., Hoffmann H. (2017). Designing future barley ideotypes using a crop model ensemble. Eur. J. Agron..

[4] Rasmusson D.C. (1991). A plant breeder’s experience with ideotype breeding. Field Crops Res..

[5] Tilman D., Balzer C., Hill J., Befort B.L. (2011). Global food demand and the sustainable intensification of agriculture. Proc. Natl. Acad. Sci. USA.

[6] Rietveld C.A., Medland S.E., Derringer J., Yang J., Esko T., Martin N.W., Westra H.J., Shakhbazov K., Abdellaoui A., Agrawal A. (2013). GWAS of 126,559 individuals identifies genetic variants associated with educational attainment. Science.

[7] Rogers E.D., Benfey P.N. (2015). Regulation of plant root system architecture: Implications for crop advancement. Curr. Opin. Biotechnol..

[8] Peleg Z., Blumwald E. (2011). Hormone balance and abiotic stress tolerance in crop plants. Curr. Opin. Plant Biol..

[9] Youssef H.M., Eggert K., Koppolu R., Alqudah A.M., Poursarebani N., Fazeli A., Sakuma S., Tagiri A., Rutten T., Govind G. (2017). VRS2 regulates hormone-mediated inflorescence patterning in barley. Nat. Genet..

[10] Alqudah A.M., Koppolu R., Wolde G.M., Graner A., Schnurbusch T. (2016). The Genetic Architecture of Barley Plant Stature. Front. Genet..

[11] Alqudah A.M., Youssef H.M., Graner A., Schnurbusch T. (2018). Natural variation and genetic make-up of leaf blade area in spring barley. Theor. Appl. Genet..

[12] Reinert S., Kortz A., Leon J., Naz A.A. (2016). Genome-Wide Association Mapping in the Global Diversity Set Reveals New QTL Controlling Root System and Related Shoot Variation in Barley. Front. Plant Sci..

[13] Digel B., Tavakol E., Verderio G., Tondelli A., Xu X., Cattivelli L., Rossini L., von Korff M. (2016). Photoperiod-H1 (Ppd-H1) Controls Leaf Size. Plant Physiol..

[14] Thirulogachandar V., Alqudah A.M., Koppolu R., Rutten T., Graner A., Hensel G., Kumlehn J., Bräutigam A., Sreenivasulu N., Schnurbusch T., Kuhlmann M. (2017). Leaf primordium size specifies leaf width and vein number among row-type classes in barley. Plant J..

[15] Ramsay L., Comadran J., Druka A., Marshall D.F., Thomas W.T., Macaulay M., MacKenzie K., Simpson C., Fuller J., Bonar N. (2011). *INTERMEDIUM-C*, a modifier of lateral spikelet fertility in barley, is an ortholog of the maize domestication gene *TEOSINTE BRANCHED 1*. Nat. Genet..

[16] Sukumaran S., Dreisigacker S., Lopes M., Chavez P., Reynolds M.P. (2015). Genome-wide association study for grain yield and related traits in an elite spring wheat population grown in temperate irrigated environments. Theor. Appl. Genet..

[17] Hedden P. (2003). The genes of the Green Revolution. Trends Genet..

[18] Marzec M., Gruszka D., Tylec P., Szarejko I. (2016). Identification and functional analysis of the HvD14 gene involved in strigolactone signaling in Hordeum vulgare. Physiol. Plant.

[19] Dockter C., Gruszka D., Braumann I., Druka A., Druka I., Franckowiak J., Gough S.P., Janeczko A., Kurowska M., Lundqvist J. (2014). Induced variations in brassinosteroid genes define barley height and sturdiness, and expand the green revolution genetic toolkit. Plant Physiol..

[20] Gupta R., Chakrabarty S.K. (2013). Gibberellic acid in plant. Plant Signal. Behav..

[21] Tong H., Xiao Y., Liu D., Gao S., Liu L., Yin Y., Jin Y., Qian Q., Chu C. (2014). Brassinosteroid Regulates Cell Elongation by Modulating Gibberellin Metabolism in Rice. Plant Cell.

[22] De Saint Germain A., Ligerot Y., Dun E.A., Pillot J.-P., Ross J.J., Beveridge C.A., Rameau C. (2013). Strigolactones Stimulate Internode Elongation Independently of Gibberellins. Plant Physiol..

[23] Daoura B.G., Chen L., Du Y., Hu Y.G. (2014). Genetic effects of dwarfing gene Rht-5 on agronomic traits in common wheat (*Triticum aestivum* L.) and QTL analysis on its linked traits. Field Crops Res..

[24] Nagano H., Onishi K., Ogasawara M., Horiuchi Y., Sano Y. (2005). Genealogy of the “Green Revolution” gene in rice. Genes Genet. Syst..

[25] Hellewell K.B., Rasmusson D.C., Gallo-Meagher M. (2000). Enhancing yield in semidwarf barley. Crop Sci..

[26] Al-Babili S., Bouwmeester H.J. (2015). Strigolactones, a novel carotenoid-derived plant hormone. Annu. Rev. Plant Biol..

[27] Kebrom T.H., Spielmeyer W., Finnegan E.J. (2013). Grasses provide new insights into regulation of shoot branching. Trends Plant Sci..

[28] Leopold A.C. (1949). The control of tillering in grasses by auxin. Am. J. Bot..

[29] Sharif R., Dale J.E. (1980). Growth-regulating substances and the growth of tiller buds in barley; effects of cytokinins. J. Exp. Bot..

[30] Liu Y., Gu D., Ding Y., Wang Q., Li G., Wang S. (2011). The relationship between nitrogen, auxin and cytokinin in the growth regulation of rice (*Oryza sativa* L.) tiller buds. Aust. J.Crop Sci..

[31] Sakamoto T., Sakakibara H., Kojima M., Yamamoto Y., Nagasaki H., Inukai Y., Sato Y., Matsuoka M. (2006). Ectopic expression of KNOTTED1-like homeobox protein induces expression of cytokinin biosynthesis genes in rice. Plant Physiol..

[32] Umehara M., Hanada A., Yoshida S., Akiyama K., Arite T., Takeda-Kamiya N., Magome H., Kamiya Y., Shirasu K., Yoneyama K. (2008). Inhibition of shoot branching by new terpenoid plant hormones. Nature.

[33] Marzec M., Muszynska A. (2015). In Silico Analysis of the Genes Encoding Proteins that Are Involved in the Biosynthesis of the RMS/MAX/D Pathway Revealed New Roles of Strigolactones in Plants. Int. J. Mol. Sci..

[34] Lo S.F., Yang S.Y., Chen K.T., Hsing Y.I., Zeevaart J.A., Chen L.J., Yu S.M. (2008). A novel class of gibberellin 2-oxidases control semidwarfism, tillering, and root development in rice. Plant Cell.

[35] Jia Q., Zhang X.Q., Westcott S., Broughton S., Cakir M., Yang J., Lance R., Li C. (2011). Expression level of a gibberellin 20-oxidase gene is associated with multiple agronomic and quality traits in barley. Theor. Appl. Genet..

[36] Ito S., Yamagami D., Umehara M., Hanada A., Yoshida S., Sasaki Y., Yajima S., Kyozuka J., Ueguchi-Tanaka M., Matsuoka M. (2017). Regulation of Strigolactone Biosynthesis by Gibberellin Signaling. Plant Physiol..

[37] Marzec M. (2017). Strigolactones and Gibberellins: A New Couple in the Phytohormone World?. Trends Plant Sci..

[38] Tong H., Jin Y., Liu W., Li F., Fang J., Yin Y., Qian Q., Zhu L., Chu C. (2009). DWARF AND LOW-TILLERING, a new member of the GRAS family, plays positive roles in brassinosteroid signaling in rice. Plant J..

[39] Wu C.Y., Trieu A., Radhakrishnan P., Kwok S.F., Harris S., Zhang K., Wang J., Wan J., Zhai H., Takatsuto S. (2008). Brassinosteroids regulate grain filling in rice. Plant Cell.

[40] Chono M., Honda I., Zeniya H., Yoneyama K., Saisho D., Takeda K., Takatsuto S., Hoshino T., Watanabe Y. (2003). A Semidwarf Phenotype of Barley uzu Results from a Nucleotide Substitution in the Gene Encoding a Putative Brassinosteroid Receptor. Plant Physiol..

[41] Gruszka D., Szarejko I., Maluszynski M. (2011). New allele of HvBRI1 gene encoding brassinosteroid receptor in barley. J. Appl. Genet..

[42] Pearce S., Vanzetti L.S., Dubcovsky J. (2013). Exogenous gibberellins induce wheat spike development under short days only in the presence of VERNALIZATION1. Plant Physiol..

[43] Pearce S., Huttly A.K., Prosser I.M., Li Y.D., Vaughan S.P., Gallova B., Patil A., Coghill J.A., Dubcovsky J., Hedden P. (2015). Heterologous expression and transcript analysis of gibberellin biosynthetic genes of grasses reveals novel functionality in the GA3ox family. BMC Plant Biol..

[44] Flintham J.E., Börner A., Worland A.J., Gale M.D. (1997). Optimizing wheat grain yield: Effects of Rht (gibberellin-insensitive) dwarfing genes. J. Agric. Sci..

[45] Boden S.A., Weiss D., Ross J.J., Davies N.W., Trevaskis B., Chandler P.M., Swain S.M. (2014). EARLY FLOWERING3 Regulates Flowering in Spring Barley by Mediating Gibberellin Production and FLOWERING LOCUS T Expression. Plant Cell.

[46] Wang Z., Cao W., Dai T., Zhou Q. (2001). Effects of exogenous hormones on floret development and grain set in wheat. Plant Growth Regul..

[47] Zheng C., Zhu Y., Zhu H., Kang G., Guo T., Wang C. (2014). Floret development and grain setting characteristics in winter wheat in response to pre-anthesis applications of 6-benzylaminopurine and boron. Field Crops Res..

[48] Patel R., Mohapatra P.K. (1992). Regulation of Spikelet Development in Rice by Hormones. J. Exp. Bot..

[49] Cao W.X., Wang Z., Dai T.B. (2000). Changes in levels of endogenous plant hormones during floret development in wheat genotypes of different spike sizes. J. Integr. Plant Biol..

[50] Wang R., Yu Z., Pan Q., Xu Y. (1999). Changes of endogenous plant hormone contents during grain development in wheat. Zuo Wu Xue Bao.

[51] Cai Q., Yuan Z., Chen M.J., Yin C.S., Luo Z.J., Zhao X.X., Liang W.Q., Hu J.P., Zhang D.B. (2014). Jasmonic acid regulates spikelet development in rice. Nat. Commun..

[52] Alqudah A.M., Schnurbusch T. (2014). Awn primordium to tipping is the most decisive developmental phase for spikelet survival in barley. Funct. Plant Biol..

[53] Alqudah A.M., Schnurbusch T. (2017). Heading Date Is Not Flowering Time in Spring Barley. Front. Plant Sci..

[54] Chhun T., Aya K., Asano K., Yamamoto E., Morinaka Y., Watanabe M., Kitano H., Ashikari M., Matsuoka M., Ueguchi-Tanaka M. (2007). Gibberellin Regulates Pollen Viability and Pollen Tube Growth in Rice. Plant Cell.

[55] Ikeda A., Ueguchi-Tanaka M., Sonoda Y., Kitano H., Koshioka M., Futsuhara Y., Matsuoka M., Yamaguchi J. (2001). slender Rice, a Constitutive Gibberellin Response Mutant, Is Caused by a Null Mutation of the SLR1 Gene, an Ortholog of the Height-Regulating Gene GAI/RGA/RHT/D8. Plant Cell.

[56] Yuo T., Yamashita Y., Kanamori H., Matsumoto T., Lundqvist U., Sato K., Ichii M., Jobling S.A., Taketa S. (2012). A SHORT INTERNODES (SHI) family transcription factor gene regulates awn elongation and pistil morphology in barley. J. Exp. Bot..

[57] Eklund D.M., Ståldal V., Valsecchi I., Cierlik I., Eriksson C., Hiratsu K., Ohme-Takagi M., Sundström J.F., Thelander M., Ezcurra I., Sundberg E. (2010). The Arabidopsis thaliana STYLISH1 Protein Acts as a Transcriptional Activator Regulating Auxin Biosynthesis. Plant Cell.

[58] Fridborg I., Kuusk S., Robertson M., Sundberg E. (2001). The Arabidopsis Protein SHI Represses Gibberellin Responses in Arabidopsis and Barley. Plant Physiol..

[59] Aya K., Ueguchi-Tanaka M., Kondo M., Hamada K., Yano K., Nishimura M., Matsuoka M. (2009). Gibberellin Modulates Anther Development in Rice via the Transcriptional Regulation of GAMYB. Plant Cell.

[60] Chen J.M., Cihlar J. (1995). Plant canopy gap-size analysis theory for improving optical measurements of leaf-area index. Appl. Opt..

[61] Jiang D., Fang J., Lou L., Zhao J., Yuan S., Yin L., Sun W., Peng L., Guo B., Li X. (2015). Characterization of a null allelic mutant of the rice NAL1 gene reveals its role in regulating cell division. PLoS ONE.

[62] Driever S.M., Lawson T., Andralojc P., Raines C.A., Parry M. (2014). Natural variation in photosynthetic capacity, growth, and yield in 64 field-grown wheat genotypes. J. Exp. Bot..

[63] Wang F.M., Huang J.F., Lou Z.H. (2011). A comparison of three methods for estimating leaf area index of paddy rice from optimal hyperspectral bands. Precis. Agric..

